# The Impact of Tobacco Smoking and Alcohol Consumption on the Development of Gastric Cancers

**DOI:** 10.3390/ijms25147854

**Published:** 2024-07-18

**Authors:** Waku Hatta, Tomoyuki Koike, Naoki Asano, Yutaka Hatayama, Yohei Ogata, Masahiro Saito, Xiaoyi Jin, Kaname Uno, Akira Imatani, Atsushi Masamune

**Affiliations:** 1 Division of Gastroenterology, Tohoku University Graduate School of Medicine, Sendai 980-8575, Miyagi, Japan; tkoike@rd5.so-net.ne.jp (T.K.); yut471@gmail.com (Y.H.); gmaps177@gmail.com (Y.O.); sitmshr@gmail.com (M.S.); kim_x_y@med.tohoku.ac.jp (X.J.); kaname@wa2.so-net.ne.jp (K.U.); aimatani@med.tohoku.ac.jp (A.I.); atsushi.masamune.d2@tohoku.ac.jp (A.M.); 2 Division of Cancer Stem Cell, Miyagi Cancer Center Research Institute, 47-1 Nodayama, Medeshima-Shiode, Natori 981-1293, Miyagi, Japan; 3 Division of Carcinogenesis and Senescence Biology, Tohoku University Graduate School of Medicine, Natori 981-1293, Miyagi, Japan

**Keywords:** gastric cancer, gastric intestinal metaplasia, tobacco smoking, alcohol drinking, alcohol consumption, polymorphism, carcinogenesis

## Abstract

Chronic infection of *Helicobacter pylori* is considered the principal cause of gastric cancers, but evidence has accumulated regarding the impact of tobacco smoking and alcohol consumption on the development of gastric cancers. Several possible mechanisms, including the activation of nicotinic acetylcholine receptors, have been proposed for smoking-induced gastric carcinogenesis. On the other hand, local acetaldehyde exposure and ethanol-induced mucosal inflammation have been proposed as the mechanisms involved in the development of gastric cancers in heavy alcohol drinkers. In addition, genetic polymorphisms are also considered to play a pivotal role in smoking-related and alcohol-related gastric carcinogenesis. In this review, we will discuss the molecular mechanisms involved in the development of gastric cancers in relation to tobacco smoking and alcohol consumption.

## 1. Introduction

Gastric cancer is the sixth most prevalent cancer and accounts for approximately 1.1 million new cases globally [1]. Its incidence has been steadily declining because of a decreased prevalence of *Helicobacter pylori (H. pylori)* infection [2], but it remains the third leading cause of death. It exhibits a high incidence in Eastern Asia and Eastern Europe and a low incidence in Northern America, Northern Europe, and Africa [1]. Endoscopic resection for early gastric cancers without lymph node metastasis is currently widely accepted as a less-invasive treatment method [3,4], and because of a relatively low prevalence of lymph node metastasis, some patients select no additional treatment even after noncurative endoscopic resection [5]. In such cases, the remnant gastric mucosa is at high risk of multi-focal gastric cancers by field cancerization.

Chronic *H. pylori* infection is considered the principal cause of gastric cancers [6]. Its eradication reduces gastric cancer incidence [7], and it improves corpus atrophy [8]. The national health insurance coverage for *H. pylori* eradication is now available in Japan for eliminating gastric cancers [9]. However, lifestyle, genetic, and epigenetic factors also affect gastric cancer progression [10], and these factors have received more attention with the prevalence of *H. pylori* eradication treatment. Tobacco smoking and alcohol drinking are well-known risk factors for esophageal squamous cell carcinoma or multiple esophageal squamous cell carcinomas [11,12,13], but the association of these factors with gastric cancers or multiple gastric cancers has accumulated evidence. For the purpose of updating the knowledge in this field, we discuss the epidemiological evidence of these associations, including functional polymorphisms of the main alcohol-metabolizing enzymes and the underlying mechanisms.

## 2. Smoking and Gastric Cancers

### 2.1. The Risk of Smoking for Gastric Cancers

Many epidemiological studies and meta-analyses have revealed the harmful effects of smoking on gastric cancers (Table 1) [14,15,16,17]. The most recent meta-analysis of 205 studies, which employed a combination of umbrella and traditional reviews, generated several findings [17]. First, the relative risk (RR) of gastric cancers compared with never smokers was 1.53 (95% confidence interval [CI], 1.44–1.62) for current smokers and 1.30 (95% CI, 1.23–1.37) for former smokers, which was almost consistent with a previous meta-analysis [16]. Second, a nonlinear association for smoking intensity was observed. The risk of gastric cancers sharply increased up to 20 tobaccos/day, the risk subsequently leveling off for a higher amount of tobaccos/day. Third, smoking duration was linearly associated with gastric cancers. The risk of gastric cancers increased linearly with smoking duration. Meanwhile, the risk of gastric cancers decreased linearly with cessation duration, reaching the level of never smokers after 30 years since cessation (RR compared with current smokers, 0.65 [95% CI, 0.44–0.95]).

A meta-analysis of 10 prospective studies observed the significant risk of gastric cancers in current smokers in both men (RR, 1.63; 95% CI, 1.44–1.85) and women (RR, 1.30; 95% CI: 1.06–1.60), with a significantly increased risk in men (the ratio of RR, 1.30; 95% CI, 1.05–1.63) [15]. Thus, a gender difference may exist in the association between smoking and gastric cancer risk, although under-reporting in women may have influenced the results [18,19].

Altogether, the association between smoking and gastric cancers is nonlinear in intensity, sharply increasing up to 20 tobaccos/day, and linear in smoking duration and cessation period. Additionally, 30 years of cessation decreases the risk of gastric cancers to the level of those who were never smokers.

### 2.2. The Risk of Smoking for Gastric Intestinal Metaplasia

According to Correa’s cascade [20], intestinal-type gastric cancer is preceded by atrophic gastritis, intestinal metaplasia, and dysplasia, following a set of sequential steps. Thus, gastric intestinal metaplasia is considered a precursor lesion of gastric cancer [21].

Many epidemiological studies have investigated the association between smoking and gastric intestinal metaplasia (Table 2) [18,22,23,24,25,26]. An earlier meta-analysis that included 5330–12,040 individuals revealed the crude odds ratios (ORs) (95% CI) of ever and current smokers for gastric intestinal metaplasia as 1.54 (1.12–2.12) and 1.27 (0.88–1.84), respectively [22]. A recent large-scale prospective cohort study that included 199,235 adults from Korea revealed that the risk of new-onset gastric intestinal metaplasia was high in both former (hazard ratio [HR], 1.17; 95% CI, 1.08–1.27) and current smokers (HR, 1.56; 95% CI, 1.45–1.68) in men, with a dose-response association, whereas no association was observed even in current smokers (HR, 0.94; 95% CI, 0.69–1.27) in women [18]. However, urinary cotinine levels revealed a positive association in both men and women, indicating that the difference may be due to gender differences in secondhand smoking or under-reporting of smoking in women [18,19]. Additionally, a more recent case-control study using the United States (US) Veterans population of 1962 individuals revealed a positive association [23]. Moreover, smoking cessation over 15 years resulted in the disappearance of statistical significance for the risk of smoking for gastric cancers compared with never smokers, further causing a negligible risk after >30 years of cessation (OR, 0.97; 95% CI, 0.59–1.59).

Collectively, smoking demonstrated a positive association with the new onset of gastric intestinal metaplasia, which is linear in smoking duration and cessation period. The risk of gastric intestinal metaplasia decreases to the level of never smokers after >30 years of cessation. This association is similar to that observed between smoking and gastric cancers.

### 2.3. The Risk of Smoking for Multiple Gastric Cancers

The remnant gastric mucosa is at a high risk for gastric cancers when gastric cancer is detected. Previous studies on endoscopic resection revealed synchronous gastric cancers accounting for 5.8–20.1% [27,28,29] and metachronous gastric cancers accounting for 9.5–13.0% in five years after endoscopic resection [30,31,32]. Thus, identifying high-risk patients for multiple cancers is important.

*H. pylori* eradication generally reduces metachronous gastric cancers [8]. Furthermore, several reports, mainly from Eastern Asia, investigated the association of smoking with multiple gastric cancers (Table 3) [31,33,34,35,36,37]. Regarding metachronous gastric cancers, two retrospective studies determined a positive association of smoking [31,35], one of which revealed a high risk of current smokers (HR, 2.65; 95% CI, 1.22–5.80), with a dose-response association [31]. In contrast, a prospective study demonstrated no significant associations in ≥40 pack-years of smoking (HR, 1.45; 95% CI, 0.80–2.66) [33].

A recent multicenter prospective study that comprised 850 patients from Japan identified ever smokers (OR, 1.93; 95% CI, 1.01–3.69) as having a high risk of synchronous gastric cancers [36], and a retrospective study from Portugal corroborates this finding [34]. In contrast, a more recent large-scale prospective cohort study that included 14,603 patients from Korea revealed no risk of synchronous gastric cancers in ever smokers (OR, 0.971; 95% CI, 0.694–1.359) [37].

Thus, the association between smoking and multiple gastric cancers is currently controversial. Further evidence should be accumulated to reach a definite conclusion.

### 2.4. Possible Mechanisms Involved in Smoking-Related Gastric Carcinogenesis

Tobacco smoke contains >5000 different molecules, 98 of which are known as carcinogens [38]. These molecules induce tumorigenesis through some possible mechanisms, including DNA adduct formation and nicotinic acetylcholine receptor (nAChR) activation, in the stomach.

Carcinogenic compounds in tobacco smoke include polycyclic aromatic hydrocarbons, nitrosamines, acetaldehyde, and aromatic amines, which can initiate tumorigenesis [39,40] mainly through DNA adduct formation, causing mutations in vital genes such as *KRAS* and *TP53* [38]. DNA adduct formation has been reported in cancer tissues of various organs, including the esophagus [41], stomach [42], and colon [43]. In the stomach, an earlier study revealed that DNA adducts were significantly higher in the gastric cancer tissues of smokers than in those of nonsmokers [42].

Nicotine is considered one of the most active components in tobacco smoke; however, its role as a direct carcinogen has been in doubt for many years [44]. The evidence of cancer cell proliferation, invasion, and metastasis by nicotine has been accumulated despite the prematurity to conclude nicotine-initiating tumorigenesis [44]. Nicotine and its metabolites bind to and activate nAChR and, to a certain extent, the β-adrenergic receptor (β-AR), thereby promoting cell proliferation, angiogenesis, and metastasis [45]. Furthermore, the metabolites of nicotine, such as cotinine, *N*′-nitrosonornicotinine, and 4-(methylnitrosamino)-1-(3-pyridyl)-1-butanone, activate nAChR, which stimulates multiple cancer-promoting signaling cascades [46,47]. Nicotine promotes cell proliferation, angioinvasion, and migration in smoking-related cancers through nAChRs, thereby activating the mitogen-activated protein kinase (MAPK)/extracellular signal-regulated kinase (ERK) pathway, phosphoinositide 3-kinase (PI3K)/AKT pathway [48], and Janus-activated kinase/signal transducer and activator of transcription signaling pathway [45]. Several pathways in gastric cancers that are activated by nicotine-nAChR, such as the PI3K/AKT pathway and ERK/5-lipoxygenase signaling cascade, have been proposed [49]. Moreover, the cyclooxygenase-2 (COX-2)-mediated mechanism for gastric cancer progression regulated by nicotine, such as the PKC-ERK1/2-activator protein-1 (AP-1)-COX-2 signaling pathway mediated by activating the β-AR, COX-2/periostin pathway and COX-2/vascular endothelial growth factor (VEGF)/VEGF receptor pathway, has been reported [49].

Meanwhile, *H. pylori* infection contributes to carcinogenesis in gastric cancers [50]. However, whether nicotine or other components in tobacco smoke act in synergy with *H. pylori* remains unclear; thus, further investigation is warranted. A previous study that evaluated the association between *H. pylori* infection and smoking, focusing on DNA methylation of marker genes in noncancerous mucosa of gastric cancer patients [51], revealed a significant association of smoking with DNA methylation levels among participants with current *H. pylori* infection, but not among those without *H. pylori* infection.

Furthermore, increasing numbers of studies in recent years have demonstrated the alternation of gastric microbiome during gastric carcinogenesis [52], but the association of microbiome and smoking-related gastric carcinogenesis remains unknown. Previous studies have shown that cigarette smoking alters the gut microbiota [53,54], and recently, a mouse model indicated that the gut microbiota dysbiosis induced by tobacco smoke plays a protumorigenic role in colorectal cancers through impaired gut barrier function and oncogenic MAPK/ERK signaling activation [55]. Thus, the role of gut microbiota in smoking-induced gastric carcinogenesis should be clarified in the future.

## 3. Alcohol Consumption and Gastric Cancers

### 3.1. Alcohol Metabolism

**Figure 1:**
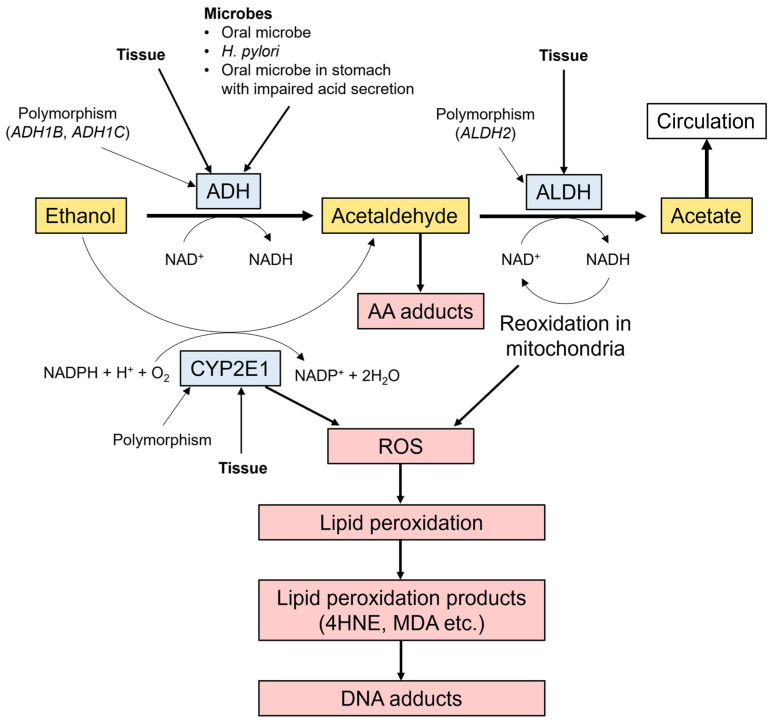
Ethanol metabolism and its role in carcinogenesis. Ethanol, metabolized by ADH, CYP2E1, and, to a much lesser extent, by catalase, is further metabolized to harmless acetate by ALDH. SNPs of *ADH1B*, *ADH1C*, *CYP2E1*, and *ALDH2* vary in ethanol and acetaldehyde exposure across individuals. Oral microbe, *H. pylori*, and the swollen oral microbe in the stomach, as well as the gastric mucosa itself possess ADH activity. Acetaldehyde promotes acetaldehyde adduct formation during ethanol metabolism, causing DNA adduct formation. Increased CYP2E1 activity causes the increased ROS generation. Both ADH and ALDH2 enzyme activity causes an increased NADH/NAD+ ratio. NADH is reoxidized to NAD+ in the mitochondria, causing further increased ROS generation. ROS-induced lipid peroxidation causes lipid peroxidation products, such as 4HNE and MDA, that interact with DNA bases to form DNA adducts. *H. pylori*, *Helicobacter pylori*; ADH, alcohol dehydrogenase; NAD, nicotinamide adenine dinucleotide; CYP2E1, cytochrome P450 2E1; ALDH, aldehyde dehydrogenase; AA, acetaldehyde; ROS, reactive oxygen species; SNPs, single nucleotide polymorphisms; 4HNE, trans-4-hydroxy-2-nonenal; MDA, malondialdehyde.

The ethanol molecule is non-carcinogenic, but the International Agency for Research on Cancer (IARC) classified its metabolite acetaldehyde as a group 1 carcinogen for humans. Alcohol dehydrogenase (ADH) oxidizes ethanol, which is further oxidized to harmless acetate by aldehyde dehydrogenase (ALDH) (Figure 1). ADH1B, ADH1C, and ALDH2, among all ADH and ALDH enzyme classes, are mainly associated with ethanol metabolism [56]. Cytochrome P450 2E1 (CYP2E1) metabolizes ethanol into acetaldehyde and generates reactive oxygen species (ROS) when alcohol consumption is high [57]. Additionally, the CYP2E1 enzyme plays a crucial role in the metabolism of nitrosamines and other carcinogenic compounds [58].

### 3.2. Genetic Polymorphisms Related to Alcohol Metabolism

#### 3.2.1. ADH1B

*ADH1B* has a variant at codon 47 (rs1229984: allele *1 = arginine and allele *2 = histidine at this codon). ADH1B*2/*2’s super-active metabolization of ethanol is approximately 40 times higher than the less active ADH1B*1/*1 [59]. ADH1B*1 is the predominant allele in most populations. ADH1B*2 allele carriers in Europe and the US account for <11%, but those in Eastern Asia are much more prevalent (67–100%) [60].

#### 3.2.2. ADH1C

*ADH1C* has a functional single nucleotide polymorphism (rs698: allele *1 = isoleucine and allele *2 = valine at codon 350). An approximately 2.5 times higher velocity has been determined for the fast ADH1C*1 allele compared with the less active ADH1C*2 allele [59,61]. The frequency of *ADH1C* genotype varies across geographic areas. The rate of the ADH1C*2 allele in the European and American populations is 31–82%, whereas that in the Asian population is <27% [60,61].

#### 3.2.3. ALDH2

*ALDH2* has a variant at codon 487 (rs671: allele *1 = glutamate and allele *2 = lysine at this codon). The ALDH2*1 allele encodes a protein with normal metabolization ability, whereas the ALDH2*2 allele encodes an inactive enzyme. Individuals with ALDH2*1/*2 demonstrated approximately 6% residual ALDH2 activity, and those with ALDH2*2/*2 exhibited no detectable activity [62]. Few populations in the US, except Asian Americans and Europeans, have the ALDH2*2 mutant allele, whereas this allele is observed in approximately 40% of the Eastern Asian population [60].

#### 3.2.4. CYP2E1

*CYP2E1* is located on the long arm of chromosome 10 and has a polymorphism in the 5′-flanking promoter region. Among many genetic polymorphisms in the *CYP2E1*, the *Rsa*I and *Pst*I polymorphism, which correspond to the C-1054T (rs2031920) and G-1293C (rs3813867) substitutions, respectively, are the most frequent polymorphisms in *CYP2E1*, causing the three following genotypes: wild-type homozygous (*c1c1*), heterozygous (*c1c2*), and variant homozygous (*c2c2*) [63,64]. The *c2* variant allele demonstrated a 10 times higher transcriptional activity, elevated protein levels, and increased enzyme activity than the wild-type *c1* allele [65]. The *c2* variant allele is presented in <10% of Caucasians, whereas the variant is shown in approximately 20–40% of the Asian population [66,67].

### 3.3. Possible Mechanisms Involved in Alcohol-Related Gastric Carcinogenesis

Ethanol itself is neither genotoxic, mutagenic, nor carcinogenic; however, available data indicate several carcinogenic mechanisms, including local acetaldehyde exposure, alcohol-induced inflammation, CYP2E1 induction, DNA methylation changes, impaired immune surveillance, and nutritional deficiencies [68,69].

#### 3.3.1. Local Acetaldehyde Exposure

Local acetaldehyde exposure is considered to play an essential role in the alcohol-related carcinogenesis of the upper gastrointestinal (GI) tract, including the stomach. Of the 10 key characteristics of carcinogens that were identified by participants at workshops convened by the IARC [70], acetaldehyde has the four following characteristics: being electrophilic, being genotoxic, altering DNA repair, and inducing oxidative stress [71]. Many oral microorganisms possess high ADH activity, enabling oxidization from ethanol to acetaldehyde (Figure 1) [71]. The ADH activity in the gastric mucosa is <1% of that in the liver and one-fifth to one-third of that in the esophagus, whereas ALDH2 activity in the gastric mucosa is about 2 to 4 times that in the esophagus [72,73]. Furthermore, the analysis of *H. pylori*-negative healthy volunteers with intragastric ethanol administration via nasogastric tube revealed that ALDH2*1/*2 carriers exhibited 5.6 times higher acetaldehyde levels than ALDH2*1/*1 carriers in gastric juice, indicating that ADH and ALDH enzymes in gastric mucosa play an important role for accumulating acetaldehyde in gastric juice [74].

*H. pylori* and other gastric microbiomes also play a crucial role in increased acetaldehyde levels in gastric mucosa. Some *H. pylori* strains possess ADH activity and produce a considerable amount of acetaldehyde when incubated with ethanol in vitro [75]. Atrophic gastritis, which is a well-known risk factor for gastric cancers, also accumulates acetaldehyde in the gastric mucosa. Oral microbial species exist in gastric juice, and metabolism by the microbiome causes high intragastric acetaldehyde levels under the condition of impaired gastric secretion, which is the consequence of atrophic gastritis [76]. Furthermore, a recent study confirmed a higher abundance of oral bacteria in gastric cancer samples than in gastric mucosa samples of cancer-predisposing stages, including atrophic gastritis and intestinal metaplasia, and indicated the key roles of oral microbes in gastric carcinogenesis [77].

For the treatment of local acetaldehyde exposure, L-cysteine may be effective since it is a semi-essential amino acid that inactivates acetaldehyde through non-enzymatic binding [78]. The slow-release L-cysteine intake has been reported to decrease acetaldehyde levels after ethanol administration both in saliva and gastric juice [74,79,80].

#### 3.3.2. Inflammation and CYP2E1 Induced by Alcohol

Chronic inflammation is known to play a key role in carcinogenesis and accelerates the oncogenic process [81]. Ethanol induces inflammation in the gastric mucosa [82]. A study using male Sprague–Dawley rats revealed that intragastric ethanol infusion caused gastric mucosal damage, which was accompanied by elevated COX-2 expression and inducible nitric oxide synthase as well as the transient activation of the redox-sensitive transcription factors, including AP-1, nuclear factor kappa-B, and MAPKs [83]. Additionally, a recent study using a mouse model revealed that alcohol consumption induced gastric inflammation, causing gastric cancers in the presence of *H. pylori* [84].

Chronic ethanol consumption induces CYP2E1 in various organs, including the liver, lung, and GI tract. CYP2E1 enzyme is neither expressed nor induced by alcohol in the epithelium of the stomach in the rat [85]. Conversely, gastric mucosa in humans expresses CYP2E1 activity, although it demonstrates lower levels than liver tissue [86]. Ethanol oxidation by CYP2E1 not only caused acetaldehyde production but ROS and reactive nitrogen species generation [57]. ROS-induced lipid peroxidation causes lipid peroxidation products, such as trans-4-hydroxy-2-nonenal, which interact with DNA bases to form DNA adducts (Figure 1) [87]. However, the data on CYP2E1 induction in gastric mucosa and acetaldehyde regulation in the gastric juice are limited.

#### 3.3.3. Gut Dysbiosis Induced by Alcohol

Previous studies have shown that alcohol consumption alters the composition of the gut microbiome [88,89], and the gut microbiome has been shown to be involved in gastric carcinogenesis [90]. Hence, the gut microbiome composition altered by alcohol consumption may contribute to gastric carcinogenesis, and further studies are warranted to verify the association.

### 3.4. The Risk of Alcohol Drinking for Gastric Cancers and Gastric Intestinal Metaplasia

The available epidemiological studies on the association between alcohol drinking and gastric cancers have been contradictory [91,92,93]. However, recent meta-analyses or a pooled analysis consistently revealed a positive association between them, at least in heavy drinkers (up to 58% increased risk of gastric cancers) (Table 4) [16,94,95,96,97,98,99,100,101,102,103]. A recent meta-analysis of 81 studies demonstrated that drinkers had an increased risk of gastric cancers (OR, 1.20; 95% CI, 1.12–1.27) [100]. A pooled analysis of 20 studies stratified into light (≤12 g/day of alcohol), moderate (>12 to 48 g/day of alcohol), heavy (>48 to 72 g/day of alcohol), and very heavy drinkers (>72 g/day of alcohol) revealed that light drinkers (OR, 1.00; 95% CI, 0.86–1.16) exhibited no increase in gastric cancer risk, whereas moderate (OR, 1.11; 95% CI, 1.01–1.23), heavy (OR, 1.26; 95% CI, 1.08–1.48), and very heavy drinkers (OR, 1.48; 95% CI, 1.29–1.70) showed an increased risk [97]. Additionally, two meta-analyses revealed that light drinkers demonstrated no elevated risk for gastric cancers (RR, 0.94–0.95) [98,102]. The pooled analysis of 20 studies also investigated the association between alcohol drinking duration and gastric cancer risk, where no consistent relation was observed between them, and hence, abstinence did not reduce the risk [97]. Some meta-analyses have proposed varying risks of gastric cancers across alcohol beverage types, but it remains controversial [95,97,100,104]. Another meta-analysis revealed a significant risk of alcohol drinking for gastric cancers in men (RR, 1.18; 95% CI, 1.06–1.32) but not in women (RR, 1.07; 95% CI, 0.95–1.19) [99].

Three studies have investigated the association between alcohol drinking and gastric intestinal metaplasia; however, the results were inconsistent [105,106,107]. A study that included 1290 patients from Poland revealed a positive association between a higher frequency of drinking vodka and gastric intestinal metaplasia (OR, 1.32; 95% CI, 1.01–1.75) [105]. However, a case-control study that included 2,084 individuals from the US revealed that even very heavy drinkers with ≥336 g/week of alcohol demonstrated no elevated risk for gastric intestinal metaplasia (OR, 1.27; 95% CI, 0.74–2.19) [107]. The risk of alcohol drinking for gastric metaplasia is expected to be not so high despite their positive association, considering the moderate association of alcohol drinking with gastric cancers. Thus, a further large-scale study is warranted to draw a definite conclusion.

Altogether, (moderate to) heavy drinkers, but not light drinkers, demonstrated an increased risk of gastric cancers. Furthermore, its risk may vary between sexes—the association between alcohol drinking and gastric intestinal metaplasia warrants further investigation.

### 3.5. Alcohol-Related Polymorphisms and Gastric Cancers

#### 3.5.1. ADH1B and ADH1C

The association of alcohol-related polymorphisms with gastric cancers has been previously investigated (Table 5) [61,108,109,110,111]. Several reports investigated the association between *ADH1B* (rs1229984) polymorphism and gastric cancers. However, there has been no study that revealed a significant association between this polymorphism and gastric cancers [108,112,113,114,115,116].

The association of *ADH1C* (rs698) polymorphism with gastric cancers is inconsistent across the studies. Two case-control studies revealed no significant results [114,117], and a case-control study from Japan demonstrated no significant association between *ADH1C* polymorphism and gastric cancers. However, compared with ADH1C*1/*1 carriers who drink 0 to <150 g/week of ethanol, ADH1C*2 allele carriers who drink ≥150 g/week of ethanol demonstrated an increased risk of gastric cancers (OR, 2.54; 95% CI, 1.05–6.17) [61].

#### 3.5.2. ALDH2

Several studies investigated the association of *ALDH2* (rs671) polymorphisms with gastric cancers, and a meta-analysis of seven case-control studies evaluated its relationship (Table 5) [109]. The meta-analysis revealed that inactive ALDH2*2 allele carriers exhibited an increased risk of gastric cancers (OR, 1.26; 95% CI, 1.04–1.52). The risk of inactive ALDH2*2 allele carriers for gastric cancers increased in moderate to heavy drinkers (OR, 1.85; 95% CI, 1.52–2.25), whereas the OR in nondrinkers or mild drinkers was 1.19 (95% CI, 1.05–1.36). Furthermore, moderate or heavy drinking increased the gastric cancer risk in inactive ALDH2*2 allele carriers (OR, 2.23; 95% CI, 1.63–3.05) more than in active ALDH2*1/*1 carriers (OR, 1.40; 95% CI, 0.98–2.01). In contrast, a recent large-scale prospective cohort study from China revealed no increased risk of gastric cancers in both ALDH2*1/*2 and ALDH2*2/*2 carriers (Table 5) [108].

#### 3.5.3. CYP2E1

Several meta-analyses on the association between *CYP2E1* polymorphisms and gastric cancers have been reported (Table 5). An earlier meta-analysis of 13 case-control studies revealed no association between *CYP2E1 Pst*I/*Rsa*I polymorphisms and gastric cancers across all included studies [110]. However, an increased risk of gastric cancers appeared in the Asian population with *c2* carriers (OR, 1.50; 95% CI, 1.16–1.94) and *c2c2* carriers (OR, 2.62; 95% CI, 1.23–5.57) when limited to high-quality scored studies. Additionally, a recent meta-analysis revealed no significant association between the analysis of *CYP2E1 Pst*I/*Rsa*I (26 studies, 9237 individuals) and *Dra*I polymorphisms (6 studies, 2342 individuals) [111]. However, a subgroup analysis of the smoking population showed that *CYP2E1 Pst*I/*Rsa*I *c2* carriers demonstrated an increased risk of gastric cancers (OR, 1.56; 95% CI, 1.14–2.15) compared with *c1c1* carriers.

### 3.6. Alcohol Drinking, Polymorphisms, and Multiple Gastric Cancers

Numerous retrospective studies have investigated the association of alcohol drinking with multiple gastric cancers; however, evaluating the accurate amount of alcohol consumption in a retrospective style is difficult. To date, there are only two prospective studies that evaluated its association, which revealed no significant results [33,118].

A recent prospective study assessed the association of *ADH1B* and *ALDH2* polymorphisms with synchronous gastric cancers [118]. The *ADH1B* polymorphism was not associated with synchronous gastric cancers, and the association of ALDH2*1/*2 with synchronous gastric cancers did not reach statistical significance. However, both the less active ADH1B*1 allele and inactive ALDH2*2 allele carriers demonstrated an increased risk of synchronous gastric cancers (OR, 1.77; 95% CI, 1.03–3.04). No studies have investigated the association of *ADH1C* or *CYP2E1* polymorphisms with multiple gastric cancers.

## 4. Combination of Smoking and Alcohol Drinking for Gastric Cancers

Several biological mechanisms encourage the combined harmful effect of smoking and alcohol drinking for gastric cancers. Chronic smoking modifies the oral flora, causing a high proportion of aerobic bacteria and yeasts possessing high ADH activity [119]. Furthermore, tobacco contains high acetaldehyde levels. Smokers and heavy drinkers independently had increased salivary acetaldehyde production by approximately 60% and 75%, respectively, compared with nonsmokers with moderate alcohol consumption in vitro. Additionally, the combination of smoking and alcohol drinking increased acetaldehyde production up to 100% both in vitro and in vivo, particularly in individuals with poor oral hygiene [120,121]. Furthermore, in vivo, salivary acetaldehyde during the tobacco smoking period in smokers increased by over 10-fold levels derived from ethanol ingestion alone [121]. CYP2E1, which metabolizes ethanol to acetaldehyde in chronic alcohol consumption, may cause metabolic activation of tobacco-derived carcinogens, such as nitrosamine [58]. Thus, explaining the harmful joint effect of smoking and alcohol drinking on developing gastric cancers is theoretically plausible.

However, the results in epidemiological investigations are inconsistent across the studies. No significant interaction in the risk of gastric cancers was found between smoking and alcohol drinking in studies from the Netherlands and Japan [91]. In contrast, a population-based study from Sweden revealed that the risk of noncardiac gastric cancers was nearly twice as high in daily smokers (HR, 1.88; 95% CI, 1.33–2.67) compared with never smokers, with no significant association between alcohol drinking and gastric cancers. However, the combined high use of tobacco (>20/day) and alcohol drinking (>5 occasions/14 days) increased the risk of noncardiac gastric cancers nearly 5-fold (HR, 4.90; 95% CI, 1.90–12.62) compared with nonsmokers [122]. Furthermore, the Continuous Update Project 2018 reported that individuals with the highest category of alcohol drinking, compared with the lowest category, had an 84% increased risk of gastric cancers in ever smokers (RR, 1.84; 95% CI, 1.43–2.36), whereas the increased risk of gastric cancers in never smokers was only 23% (RR, 1.23; 95% CI, 1.03–1.46) [123].

## 5. Conclusions and Future Direction

As we have discussed above, smoking and heavy alcohol consumption are contributing factors to the development of gastric cancers, and smoking is associated with gastric intestinal metaplasia. Furthermore, smoking cessation reduces the risk of gastric cancers and gastric intestinal metaplasia to almost no risk in the cessation period of ≥30 years. Thus, patients, doctors, and health policy leaders have to know that smoking should be avoided and alcohol consumption should be kept to a moderate level to prevent gastric cancer. In Japan, the government has designated the week starting from World No Tobacco Day as No Smoking Week, and additionally, students are taught about the risks of tobacco smoking and alcohol consumption on their health in schools. In Europe and the US, some projects, such as the European School Survey Project on Alcohol and Other Drugs [124,125] and the Life Skills Training Program [126], have been developed. These projects are supported from the perspective of preventing gastric cancer based on the results shown in this review. Meanwhile, some results were not consistent across the studies in this review; thus, further studies remain warranted in this field. Furthermore, since *H. pylori* is the principal cause of gastric cancers and the gut microbiota dysbiosis induced by tobacco smoke demonstrates a protumorigenic role in colorectal cancers [55], and since ample evidence suggests the involvement of gut microbiota in gastric carcinogenesis [90], characterizing the role of smoking under the condition of *H. pylori* infection and atrophic gastritis, especially from the perspective of gut microbiota dysbiosis, is needed.

## Figures and Tables

**Table 1:** Recent meta-analyses evaluating the association between smoking and gastric cancers.

Author, Years	No. of Studies Included	No. of Cases	Smoking Status	Risk of Gastric Cancer ^†^
Ferro et al., 2018 [14]	20 case-control studies 23 case-control studies (IPD)	– ^§^ 36,020	Ever Ever	OR ^‡^, 1.28 (1.17–1.41) OR ^‡^, 1.20 (1.09–1.32)
Li et al., 2019 [15]	9 prospective cohort and 1 nested case-control studies	3,381,345	Current (men) Current (women)	RR ^‡^, 1.63 (1.44–1.85) RR ^‡^, 1.30 (1.06–1.60)
Poorolajal et al., 2020 [16]	77 studies	– ^§^	Current Former	OR ^‡^, 1.61 (1.49–1.75) OR ^‡^, 1.43 (1.29–1.59)
Rota et al., 2024 [17]	129 case-control and 76 cohort studies	677,040 (gastric cancer cases)	Current Former 10 tobaccos/day 20 tobaccos/day 20 years of duration 40 years of duration 10 years of cessation 20 years of cessation 30 years of cessation	RR ^‡^, 1.53 (1.44–1.62) RR ^‡^, 1.30 (1.23–1.37) RR ^‡^, 1.45 (1.36–1.54) RR ^‡^, 1.69 (1.55–1.84) RR ^‡^, 1.31 (1.25–1.37) RR ^‡^, 1.72 (1.56–1.89) RR ^¶^, 0.87 (0.76–0.98) RR ^¶^, 0.75 (0.58–0.96) RR ^¶^, 0.65 (0.44–0.95)

^†^ Parentheses indicate a 95% confidence interval. ^‡^ Compared with never smokers. ^¶^ Compared with current smokers. ^§^ Number of cases was not shown. IPD, individual participant data; OR, odds ratio; RR, relative risk.

**Table 2:** Reports evaluating the association between smoking and gastric intestinal metaplasia.

Author, Years	Study Design, Countries	No. of Cases	Smoking Status	Risk of Gastric Intestinal Metaplasia ^†,‡^
Morais et al., 2014 [22]	Meta-analysis of 9–13 studies	12,040 5330	Ever Current	OR, 1.54 (1.12–2.12) OR, 1.27 (0.88–1.84)
Gomez et al., 2017 [24]	Case-control, US	639	Tobacco use	OR, 1.73 (1.18–2.55)
Kim et al., 2019 [18]	Prospective cohort, Korea	199,235	Current (men) Former (men) Current (women) Former (women)	HR, 1.56 (1.45–1.68) HR, 1.17 (1.08–1.27) HR, 0.94 (0.69–1.27) HR, 0.94 (0.64–1.39)
Zullo et al., 2021 [26]	Case-control (prospectively collected data), Italy	1311	Smoking habit	OR, 1.37 (0.67–2.79)
Kligman et al., 2022 [25]	Case-control, US	2685	Ever	OR, 1.68 (1.30–2.15)
Thrift et al., 2022 [23]	Case-control, US	1962	Current Former ≤25 years of duration 25.01–40 years of duration >40 years of duration ≤15 years of cessation 15.01–30 years of cessation >30 years of cessation	OR, 2.05 (1.47–2.85) OR, 1.40 (1.02–1.93) OR, 1.16 (0.80–1.67) OR, 2.00 (1.43–2.80) OR, 1.86 (1.30–2.67) OR, 1.65 (1.12–2.43) OR, 1.34 (0.90–2.00) OR, 0.97 (0.59–1.59)

^†^ Parentheses indicate a 95% confidence interval. ^‡^ Compared with never smokers. OR, odds ratio; HR, hazard ratio; US, the United States.

**Table 3:** Reports evaluating the association between smoking and multiple gastric cancers in the multivariate analysis.

Author, Years	Study Design, Countries	Synchronous/ Metachronous	No. of Cases	Smoking Status	Risk of Multiple Gastric Cancers ^†^
Mori et al., 2016 [33]	Prospective cohort, Japan	Metachronous	594	≥40 pack-years	HR ^‡^, 1.45 (0.80–2.66)
Ami et al., 2017 [31]	Retrospective cohort, Japan	Metachronous	539	Current Former ≥20 pack-years	HR ^‡^, 2.65 (1.22–5.80) HR ^‡^, 1.16 (0.76–1.76) HR ^‡^, 1.51 (1.03–2.24)
Brito-Gonçalves et al., 2020 [34]	Retrospective cohort, Portugal	Synchronous Metachronous	230	Ever Ever	OR ^‡^, 3.64 (1.07–12.40) OR ^‡^, 0.47 (0.07–3.40)
Ishioka et al., 2021 [35]	Retrospective cohort, Japan	Metachronous	525	≥30 pack-years	RR ^¶^, 1.84 (1.20–2.84)
Hatta et al., 2023 [36]	Prospective cohort, Japan	Synchronous	850	Current Former ≥20 pack-years	OR ^‡^, 2.33 (1.14–4.78) OR ^‡^, 1.76 (0.90–3.43) OR ^‡^, 2.26 (1.12–4.57)
Song et al., 2024 [37]	Prospective cohort, Korea	Synchronous	14,603	Ever	OR ^‡^, 0.971 (0.694–1.359)

^†^ Parentheses indicate a 95% confidence interval. ^‡^ Compared with never smokers. ^¶^ Compared with <30 pack-years of smoking. HR, hazard ratio; OR, odds ratio; RR, relative risk.

**Table 4:** Primary results of meta-analyses or a pooled analysis evaluating the association between alcohol drinking and gastric cancers.

Author, Years	No. of Studies Included	No. of Cases	Alcohol Drinking Status	Risk of Gastric Cancer ^†^
Deng et al., 2021 [100]	68 case-control and 13 cohort studies	167,033 (gastric cancer cases	Drinkers	OR ^‡^, 1.20 (1.12–1.27)
Rota et al., 2017 [97]	20 case-control studies (a pooled analysis)	35,005	Light drinkers Moderate drinkers Heavy drinkers Very heavy drinkers >0 to 20 years of duration >20 to 40 years of duration >40 years of duration >0 to 5 years of quitting >5 to 10 years of quitting >10 years of quitting	OR ^‡^, 1.00 (0.86–1.16) OR ^‡^, 1.11 (1.01–1.23) OR ^‡^, 1.26 (1.08–1.48) OR ^‡^, 1.48 (1.29–1.70) OR ^‡^, 1.02 (0.84–1.23) OR ^‡^, 1.28 (1.08–1.51) OR ^‡^, 1.13 (0.97–1.33) OR ^¶^, 1.93 (1.39–2.68) OR ^¶^, 1.00 (0.66–1.53) OR ^¶^, 0.84 (0.56–1.25)
Bae et al., 2021 [99]	27 prospective cohort studies 13 prospective cohort studies 7 prospective cohort studies	–	Alcohol drinking Alcohol drinking (men) Alcohol drinking (women)	RR ^§^, 1.13 (1.04–1.23) RR ^§^, 1.18 (1.06–1.32) RR ^§^, 1.07 (0.95–1.19)

^†^ Parentheses indicate a 95% confidence interval. ^‡^ Compared with no drinkers. ^¶^ Compared with current drinkers. ^§^ Highest vs. lowest level of alcohol drinking. OR, odds ratio; RR, relative risk.

**Table 5:** Reports evaluating the association between alcohol-related polymorphisms and gastric cancers.

Author, Years	Study Design, Countries	No. of Cases	Polymorphism Status	Risk of Gastric Cancer ^†^
*ADH1B* (rs1229984) **^‡^**				
Im et al., 2022 [108]	Prospective cohort, China	150,722	ADH1B*1/*2 men ADH1B*1/*2 women ADH1B*2/*2 men ADH1B*2/*2 women	HR ^¶^, 0.98 (0.88–1.09) HR ^¶^, 1.05 (0.90–1.23) HR ^¶^, 0.98 (0.88–1.09) HR ^¶^, 1.03 (0.89–1.20)
*ADH1C* (rs698) **^‡^**				
Hidaka et al., 2015 [61]	Case-control, Japan	914	ADH1C*2 allele carriers ADH1C*2 allele carriers who drink ≥150 g/week of ethanol	OR ^§^, 0.79 (0.51–1.22) OR ^a^, 2.54 (1.05–6.17)
*ALDH2* (rs671)				
Joo Kang et al., 2021 [109]	Meta-analysis of 7 studies	8194	ALDH2*2 allele carriers ALDH2*2 allele carriers in moderate/heavy drinkers ALDH2*2 allele carriers in no/mild drinkers	OR ^b^, 1.26 (1.04–1.52) OR ^b^, 1.85 (1.52–2.25) OR ^b^, 1.19 (1.05–1.36)
Im et al., 2022 [108]	Prospective cohort, China	150,722	ALDH2*1/*2 men ALDH2*1/*2 women ALDH2*2/*2 men ALDH2*2/*2 women	HR ^b^, 1.05 (0.93–1.19) HR ^b^, 1.17 (0.99–1.38) HR ^b^, 0.84 (0.55–1.26) HR ^b^, 1.49 (0.99-2.25)
*CYP2E1* (rs2031920, rs3813867)				
Boccia et al., 2007 [110]	Meta-analysis of 13 studies	4820	*c2* carriers *c2c2* carriers *c2* carriers *c2c2* carriers	OR ^c^, 0.97 (0.79–1.18) OR ^c^, 1.36 (0.82–2.25) OR ^c, d^, 1.50 (1.16–1.94) OR ^c, d^, 2.62 (1.23–5.57)
Zhang et al., 2016 [111]	Meta-analysis of 26 studies	9237	*c2* carriers *c2* carriers in smoking population	OR ^c^, 0.96 (0.82–1.12) OR ^c^, 1.56 (1.14–2.15)

^†^ Parentheses indicate a 95% confidence interval. ^‡^ Only the largest study is shown in this table. ^¶^ Compared with ADH1B*1/*1 men/women. ^§^ Compared with ADH1C*1/*1 carriers. ^a^ Compared with ADH1C*1/*1 carriers who drank 0 to <150 g/week. ^b^ Compared with ALDH2*1/*1 men/women. ^c^ Compared with *c1c1* carriers. ^d^ Limited to high-quality scored studies in the Asian population. *ADH*, *alcohol dehydrogenase*; HR, hazard ratio; OR, odds ratio; *ALDH*, *aldehyde dehydrogenase*; *CYP2E1*, *cytochrome P450 2E1*.
